# A qualitative exploration of a financial inclusion service in an English foodbank

**DOI:** 10.1177/17579139231180755

**Published:** 2023-07-12

**Authors:** S. Hanson, B Porter

**Affiliations:** School of Health Sciences, University of East Anglia, Norwich, UK; School of Health Sciences, University of East Anglia, Norwich, UK

**Keywords:** foodbank, financial inclusion, poverty

## Abstract

**Aims:**

Foodbanks provide emergency food provision. This need can be triggered by a change in circumstance or a crisis. Failures in the social security safety net are the most significant driver for hunger in the UK. There is some evidence that an advisory service which runs alongside a foodbank is more effective in reducing emergency provision and the duration and severity of hunger. The ‘Making a Difference’ project at an English foodbank is a pilot scheme aiming to increase financial resilience in their service users. From summer 2022, they introduced new advice worker roles, in partnership with Shelter [Housing advice] and Citizen’s Advice [General, debt and benefits advice], aiming to pre-empt the need for foodbank use, to triage the financial needs of service users and refer appropriately to reduce repeat visits to the foodbank.

**Methods:**

This qualitative study involved in-depth interviews with four staff and four volunteers to evaluate barriers, facilitators and potential friction points in referrals and partnership working.

**Findings:**

Our data were analysed thematically into four themes: Holistic needs assessment; Reaching seldom heard communities; Empowerment; The needs of staff and volunteers. Two case studies illustrate the complexity of people’s needs.

**Conclusion:**

A financial inclusion service operating within foodbanks giving housing, debt and benefits advice shows some promise in reaching people in crisis at the point of need. Based within the heart of a community, it appears to meet the complex needs of very vulnerable people who may have found mainstream support services inaccessible. This asset-based approach with the foodbank as a trusted provider enabled joined up, compassionate, holistic, and person-centred advice quickly cutting across multiple agencies, reaching underserved and socially excluded clients. We suggest that supportive services are needed for volunteers and staff who are vulnerable to vicarious trauma from listening and supporting people in crisis.

## Background

Material poverty, and its root causes, such as the use of food banks is a major public health concern in the UK. The ‘State of Hunger’^1^ provides clear links between the growth in need for emergency food (such as from foodbanks) and changes inherent in the UK social security system. This is often compounded by adverse life experiences and ill-health, which worsen people’s financial situation, such as extra expenses or through undermining people’s capacity to navigate the benefit system. In some cases, benefit problems clearly also exacerbate health conditions.^2^

Evidence given to the All Parliamentary Group on Hunger stated that, ‘*the more support made available to people during their first visit to a food bank, the shorter the period of time they are likely to be hungry’*,^3^ suggesting that advice workers could reduce the duration and severity of hunger so they no longer need to rely on emergency foods.^1^ Signposting, for example, giving information about other local organisations, is not enough, often resulting in return visits to the foodbank.^3^ This is likely to be because people’s issues are complex and need a more holistic approach.

The ‘Making a Difference’ project is a pilot advisory scheme in foodbanks in the east of England with advisers from Shelter and Citizen’s Advice. The foodbank is part of The Trussell Trust a national foodbank provider. This research aimed to capture any learnings from operationalising the scheme that might affect successful implementation of this service more widely and to create lived-experience examples to illustrate the needs and particular issues of clients in the current climate.

## Methods

We gained Institutional ethics from The Faculty of Medicine and Health, University of East Anglia prior to data collection (Ref: ETH2122-2144). The research was promoted by the foodbank, and staff and volunteers who were interested in taking part contacted the researchers for information. In total, four paid staff (advice workers) and four volunteers consented to take part. We used semi-structured interviews encouraging people to share their experiences in an in-depth way within its real-life context.^4^ We analysed our data thematically^5^ and generated lived-experience cases to give us real-world examples.^6^

Interviews were completed both face-to-face and online from July to October 2022. They were digitally voice recorded, transcribed and identifying information was removed. A copy of the findings was given to participants for the opportunity to revise their responses.

## Findings

In 2019/2020, the foodbank supported 12,577 people (it was 8905 in 2015) with 300 referral agencies, with Citizen’s Advice as the largest (817 referrals). The top reason for referrals being: Low income (3,746), Benefit changes (2,324), Benefit delays (2,276), Debt (2,094), Homeless (511).

The analysis of our interviews pointed to four key themes:

### Holistic complex needs assessment

A person-centred approach was seen as essential and variously described, such as reaching out with kindness.

*Having someone who cares enough to call them by their name, that just opens the door for us to be able to help in other ways. . . it is part of the holistic approach to poverty*. (Staff 4)

Clients had very complex needs that cut across many different services.

*That’s the stuff that really is important in people’s lives, how to navigate the benefit system, the housing systems, to ensure that they do have a reasonable standard of living. You have to work with the whole person and all that they’re coming with*. (Staff 5)

*I’m really passionate about helping people on that day, and that wrap around support. . . being able to give people that holistic advice is great. (Volunteer 4)*

*We’re dealing with many people that are really on the edges if you like of financial exclusion, we’re also seeing lots of people with poor mental health issues, people that basically social services are failing.* (Staff 3)

There was trauma as well as complexity.

*You try and sort it out there and then because then that’s the best thing for the clients. They don’t have to tell this story about five times to different organisations, it can re-traumatise them to think about things they don’t wanna think about*. (Staff 2)

‘Social Prescribing’ featured in all our interviews and this was more than ‘signposting’.

*Social prescribing. We call it hand holding ‘cause, it’s almost like you need to hold their hand and say it’s OK, come with me, I’ll lead you to this free group, I will lead you to this contact . . . to find the right people and they have to be able to trust me.* (Staff 4)

### Reaching seldom-supported communities

The ‘Making a Difference project’ is situated in the heart of very deprived communities (20% most deprived in England). Our data suggest that the service is reaching people who ‘fall through the gaps’.

*They’re not, like the normal Citizens Advice type of client. I wouldn’t have got them if I’ve just been at Citizen’s Advice. They are like an undercurrent that no one wants to truly talk about . . . but I am talking about the ones that we should be there to help*. (Staff 3)

*I think a lot of people can’t just get their head around seeing Citizens Advice. I think the foodbank has enabled more people to get the help that they do need*. (Volunteer 3)

*It feels like our service is a good way to access really vulnerable people that might not come otherwise*. (Staff 2)

Local knowledge helped to streamline and facilitate access to other organisations.

*You can get a sense of common themes of what’s happening . . . and building those community partnerships . . . you need to get local services involved that have local knowledge*. (Staff 2)

*It really is kind of a community hub. Sometimes people will come in just because they know the Shelter advisor* [housing advice] *is there and not actually, you know, trying to access the foodbank*. (Volunteer)

### Empowerment

Part of empowering clients was treating them with dignity and sensitivity in often distressing circumstances.

*It’s really important that you don’t just do something for somebody that they’re part of the journey and they become informed and empowered as a result of it as well . . . I’m here. This is my role. You’re in control. You tell me what you want and need. I’m basically working for you.* (Staff 1)

*Building the relationships with the volunteers is absolutely key because they’re the ones with the experience and knowledge around their client base and, the same people come back every week to these hubs and they know them, it’s really impressive.* (Staff 5)

### The needs of staff and volunteers

Staff and volunteers found the work very rewarding, but this is a frontline and very challenging landscape.

*It’s quite disheartening sometimes . . . how this can actually impact your own health as well, you know like kind vicarious trauma and things like that because it can be really draining to do that, but then there’s just the amazing feeling that you’ve helped as well*. (Staff 2)

*I suppose there’s a degree of satisfaction of helping people but that is tinged with lots of sadness as to why they’re there and how they’re* there. (Volunteer 2)

## Discussion

By operating in the heart of the community, and delivering housing, debt, benefit and other advice at the point of need, the foodbank is taking an asset-based approach^7^ with local knowledge helping to streamline and facilitate access to other organisations quickly. Our findings suggest that this approach is reaching very vulnerable people who often miss out on support, such as state benefits. For example, in 2021, on average only 54% of eligible families received Healthy Start vouchers therefore missing out on this important benefit^8^ which has long-term implications for health. Pension credit is a ‘top-up’ benefit (up to £3,300 per year available to people on low income). Yet uptake has stagnated at around 60% over the last 10 years; £2.8 billion has gone unclaimed every year since 2009/10 and importantly low uptake of pension credit actually costs the government £4 billion in increased National Health Service (NHS) and social care spending.^9^

Our findings suggest that an advice service offering holistic support in a foodbank, at the point of acute need shows promise in effectively compensating the most vulnerable people with complex needs who may have previously found advice services inaccessible and who are too often socially excluded from opportunities and services that could support them. This asset-based approach with the foodbank as a trusted provider enabled joined up, compassionate, holistic and person-centred advice, quickly cutting across multiple agencies, reaching underserved and socially excluded clients. This approach also represents an opportunity to broaden the skills of staff and volunteers by empowering them to support people in crisis in a comprehensive way. We also suggest supportive services for volunteers and staff who are vulnerable to vicarious trauma from a frontline role listening and supporting people in crisis in a very challenging landscape.^10^

## Supplemental Material

sj-docx-1-rsh-10.1177_17579139231180755 – Supplemental material for A qualitative exploration of a financial inclusion service in an English foodbank Supplemental material, sj-docx-1-rsh-10.1177_17579139231180755 for A qualitative exploration of a financial inclusion service in an English foodbank by S. Hanson and B Porter in Perspectives in Public Health

## References

[1] Sosenko F Littlewood M Bramley G , et al. State of Hunger: a study of poverty and food insecurity in the UK. Project report, The Trussell Trust, November 2019.

[2] Trussell Trust. State of Hunger, Building the Evidence on Poverty, Destitution, and Food Insecurity in the UK. Available online at: https://www.trusselltrust.org/wp-content/uploads/sites/2/2021/05/State-of-Hunger-2021-Report-Final.pdf (last accessed 31 May 2022)

[3] Feeding Britain. Pathways from Poverty. Available online at: https://feedingbritain.org/what-works-centre/pathways-from-poverty/ (last accessed 29 June 2022)

[4] Muellmann S Brand T Jürgens D , et al. How many key informants are enough? Analysing the validity of the community readiness assessment. BMC Res Notes 2021;14:85. 33750436 10.1186/s13104-021-05497-9 PMC7941941

[5] Braun V Clarke V . Using thematic analysis in psychology. Qual Res Psychol 2006;3:77–101.

[6] Yin RK . Case study research: design and methods. 4th edn. Thousand Oaks, CA: Sage; 2009.

[7] Hopkins T Rippon S . Head, hands and heart: asset-based approaches in health care. London: The Health Foundation; 2015.

[8] Sustain. Healthy Start: Estimated Loss to Families in 2021. Available online at: https://www.sustainweb.org/foodpoverty/cash_shortfall_local_map/ (last accessed 24 August 2022)

[9] Independent Age. Credit Where It’s Due: A briefing on Low Uptake of Pension Credit. Available online at: https://www.independentage.org/sites/default/files/2020-10/Pension%20credit%20briefing_5.pdf (last accessed 31 August 2022)

[10] Howlett SL Collins A . Vicarious traumatisation: Risk and resilience among crisis support volunteers in a community organisation. S Afr J Psychol 2014;44:180–90.

