# Supplementary material for: A qualitative exploration of a financial inclusion service in an English foodbank
Source: Perspect Public Health. 2023 Jul 12;145(1):42–4. doi: 10.1177/17579139231180755 (PMC11800711; doi:10.1177/17579139231180755)
Supplement: sj-docx-1-rsh-10.1177_17579139231180755 – Supplemental material for A qualitative exploration of a financial inclusion service in an English foodbank [file sj-docx-1-rsh-10.1177_17579139231180755.docx]

Case studies for supplementary information

Case study I

**Rosie is a** woman with two very young children and has a chronic, life-limiting health condition and mobility issues who is registered disabled. Her particular circumstance was a rehousing into temporary accommodation due to domestic abuse. This relocation took her away from any social support and the emergency hotel accommodation meant that she had no access to cooking facilities. In addition to her emergency situation, the unsuitable accommodation (for her circumstances and her disability) meant that she had unnecessary costs due to having to eat out three times a day (A minimum of 42 meals). She attended the foodbank to get an emergency food parcel and was seen by a specialist adviser on the same day. She had less than £50 to last the week.

The outcomes of this were emergency food and kettle kits to use in the hotel to help with her food crisis. A referral was made to a local Assistance Scheme due to the additional costs she had from fleeing from domestic violence (for example buying a double buggy so that she could walk to appointments). Additionally, she was given specialist housing advice which resulted in a move to accommodation the following day that suited her disability and mobility needs and enabled her to be able to cook for her children. Emergency food was arranged for the new accommodation. Staff, using their local contacts also took her to a place in Norwich where she was able to choose new clothes and started the process of a new bank account to enable her to receive statutory benefits.

*If I hadn't been at the food bank, she could have just been wandering around the streets or whatever. She didn't know where to go. She was just waiting on a phone call. That's all she was waiting on from the council when she first came into the foodbank. She's like, they still haven't rung me, they still haven't rung me. I was like, don't worry, they’ll ring you again. And the volunteers were like, let's get a cup of tea. Chocolate for the kids and crisps and we will all sort this*. (Specialist adviser)

Case study II

**Peter is a** single male who shares custody of his two teenage children after having recently had a relationship breakdown. He had worked as a builder but had also recently lost his job during the same time as he was having to move out of the family home and look for rented accommodation. He viewed himself as a typical masculine bloke, who had worked hard his entire life and managed to generally get by. He had never accessed foodbanks or services like that before.

When he first approached the foodbank, he could not bring himself to come inside and sat in his van, in tears and just absolutely devastated he was even there. On this visit, the volunteers served him from his van, offering him a cup of tea and some breakfast. The volunteer could clearly see his distress and wanted to see if someone could get to the bottom of what was really going on for him, rather than just sending him off with a food parcel and seeing him again next week.

On this occasion, the advice worker based at the foodbank sat with the man in his van talked to him about his situation and found different ways they could help. He was in debt of £50 on his energy meter and could not afford to run a hot bath for his teenage children when they came to stay. He was able to receive an energy meter top-up and have the debt paid off, and the advice worker helped to figure out what benefits he would be entitled to. He was also able to get food supplied for his beloved dog from the foodbank.

For a few more weeks he continued to come and collect food parcels. This man still visits the foodbank, but not as a client or to use the services. He comes regularly to have breakfast in the community space and chat with the volunteers, sometimes he brings his dog and the kitchen spoil the dog with bits of sausage and bacon.
